# Operative Versus Nonoperative Management of Hip Fractures in Older Adults: Clinical Outcomes and Palliative Alignment

**DOI:** 10.7759/cureus.99313

**Published:** 2025-12-15

**Authors:** Mendel Shloush, Brayden Tolman, Menachem Friedman, Ana Viamonte Ros

**Affiliations:** 1 Orthopedic Surgery, Herbert Wertheim College of Medicine, Florida International University, Miami, USA; 2 Anesthesiology, Herbert Wertheim College of Medicine, Florida International University, Miami, USA; 3 Anatomy, Florida International University, Miami, USA; 4 Palliative Care, Herbert Wertheim College of Medicine, Florida International University, Miami, USA

**Keywords:** lower extremity fractures, nonoperative management, older adults, operative management, palliative care, quality of life, shared decision-making

## Abstract

Hip fractures in older adults present considerable challenges in health outcomes and management. Deciding between surgical and nonsurgical treatment is especially complex in patients with serious comorbidities, those receiving palliative care, or individuals nearing the end of life. This review compares operative versus nonoperative treatment of hip fractures, discussing the implications related to mortality, quality of life (QOL), and harm versus burden. While surgical treatment is often considered the standard of care, it carries significant risks in frail older adults, such as postoperative infections, pain, and anesthesia-related complications. This review explores whether nonoperative treatment for hip fractures in frail older adults or those nearing the end of life can be a viable alternative without increasing harm. Evidence suggests that a comparable or even superior quality of life can be achieved nonoperatively in these patients through pain management and palliative care. While nonoperative management is linked to higher short-term mortality, it may better align with older patients’ goals by emphasizing comfort and quality of life. These findings underscore the importance of an individualized, patient centered, and shared decision-making approach when caring for these patients. Considering patients’ goals, frailty, cognitive status, and prognosis is key to optimizing outcomes. By adopting a more holistic approach, palliative medicine will have increasing importance in supporting older adults who suffer from hip fractures.

## Introduction and background

One of the fastest-growing groups in the United States today is the older adult population. Approximately 16.8% of adults in the United States are aged 65 years or older, and that percentage is projected to be higher than 20% by 2030 [1]. Traumatic injuries occur quite frequently in older adults, with almost half of all reported traumatic injuries occurring in geriatric patients [2]. Among the different injuries sustained in older adults, hip fractures are considered quite serious due to potential complications. Such injuries are associated with significant illness and mortality rates, along with deterioration of patients' quality of life [3,4]. Many patients suffering from hip fractures have reduced mobility, often requiring admission to care facilities and resulting in a decreased quality of life [5]. In the United States alone, between 260,000 and 300,000 patients are hospitalized for hip fractures each year. Experts believe that this number can possibly increase to over 500,000 annually by 2040 [6]. In terms of mortality, studies have shown that 2-14% of injured older patients die during their hospital stay, while 14-36% die within a year after surgery [7]. Among patients with neurocognitive disorders, the mortality rate at six months postoperative may be as high as 55% [3]. Furthermore, of those patients who have successful surgical procedures, only 50% will return to their pre-injury level of independence, with nearly 20% requiring relocation to long-term care [8].

There are the following two broad treatment routes for patients with hip fractures: operative and nonoperative management. Of these, the most common is surgery, while nonsurgical management is only pursued in about 10% of patients [4]. The goals of surgery are to increase functional status and return a patient to their pre-fracture state [4]. However, there are many associated risks of surgery, especially among older, frail patients, such as those with increased fatigue and reduced tolerance to treatment, who have pre-existing comorbidities. Complications like delirium or anesthesia-related issues may occur, and additional impairment in cognitive function may occur in neurocognitive disorder patients who undergo surgery [9,10]. Nonsurgical management includes pain management and early mobilization protocols [8]. While this treatment plan is less often pursued, it may be the more appropriate option for certain high-risk patients, particularly frail older adults. In order to decide on the best choice for each patient, all parameters must be carefully considered, such as the patient's comorbidities, goals of care, and quality of life. This approach enables the determination of which treatment option best suits individual patients and the development of treatment plans accordingly [11,12].

Palliative care is an interdisciplinary approach that focuses on enhancing the quality of life for patients who suffer from severe conditions. This is achieved through pain management, discussion of goals of care, and care coordination, which help improve patients' physical, social, emotional, and spiritual needs [8,13]. Additionally, palliative care supports family members and caregivers as needs arise during patient care [14,15]. Although classically associated with end-of-life care, palliative care can be beneficial early on in the disease course, regardless of prognosis [16]. Major organizations, including the Joint Commission on Accreditation of Healthcare Organizations, the National Academy of Medicine, and the World Health Organization, have called for improvements in palliative care services, underscoring its importance [4]. As a result, hospitals have dedicated inpatient palliative care services to improve accessibility [17]. When treating older patients with hip fractures, it is critical to implement palliative care services in order to enable open communication, discuss prognosis, and address caregiver needs [18,19].

In this concept review, operative and nonoperative treatment will be compared regarding quality of life, survival, and harm. Our goal is to determine whether nonoperative treatments are effective, or even preferred, for some patients with hip fractures by demonstrating improved quality of life without exposure to surgical-related harm.

Although surgery was, and still is, the standard of care in hip fractures, there is growing evidence to support nonoperative treatment as an alternative when caring for older, frail patients with limited life expectancy [3]. In fact, this aligns well with recent recommendations from the American Geriatrics Society and the American College of Surgeons, which called for incorporating palliative care assessments and baseline frailty screening for older surgical candidates [16]. We hope to contribute to the growing body of literature by highlighting the need for a holistic, patient-centered approach in the management of hip fractures based on key variables, including goals of care, cognitive status, and frailty [20-22].

In evaluating treatment options for older adults with hip fractures, it is important to consider not only survival outcomes but also the balance of harm versus burden. Harm refers to the direct negative consequences of an intervention, such as surgical complications or cognitive decline, while burden encompasses the overall impact of treatment on a patient’s quality of life. For frail or end-of-life patients, there are cases where nonoperative management may better align with a patient’s goals by minimizing harm and reducing the burden of recovery, emphasizing comfort and quality of life over more aggressive interventions.

Literature selection approach

A targeted literature search was performed using the PubMed database to identify relevant studies on operative and nonoperative management of hip fractures in older adults. Keywords included combinations of “hip fracture,” “older adults,” “frailty,” “nonoperative management,” “operative management,” “quality of life,” “palliative care,” “shared decision-making,” “end-of-life care,” and “mortality.” These terms were used individually and in combination to capture studies addressing outcomes, quality of life, and patient-centered care in this population. No formal statistical synthesis was performed, as this review is conceptual and narrative in nature, focusing on the interpretation and integration of existing evidence rather than quantitative comparison. Given the narrative nature of this review, the selection of literature was based on conceptual relevance rather than on systematic search parameters.

## Review

Survival

Fragility fractures are a major concern in older adults due to the resulting increase in morbidity and mortality [23]. Hip fractures are of particular concern in the older population. These fractures often occur in nursing homes or while on hospice, a time when most of these patients are in the beginning or active stages of dying [4,24]. When considering that the one-year mortality rate for older adults with a hip fracture is between 12% and 36% and only one-third of patients return to pre-fracture status [23], it is important to evaluate the true benefit that surgery can have in those nearing the end of their lives [4].

In a study comparing operative and nonoperative management of proximal femoral fractures in a population with a mean age of 88 years, the 30-day mortality rate was 25% in the operative management group and 83% in the nonoperative management group [3]. At six months, those rates increased to 48% and 94%, respectively [3]. However, although mortality was higher in the nonoperative cohort, other factors should be considered. Of those patients who died during the study, proxies and practitioners involved in the nonoperative cases reported greater satisfaction with this choice compared to those involved in the operative management cohort [3]. Additionally, 51% of proxies in the nonoperative group rated the quality of dying as good-almost-perfect in the nonoperative group, whereas 62% of proxies in the operative group rated it as intermediate [3]. Thus, nonoperative treatment of hip fractures appears to be a viable alternative to surgery for select older patients, as determined by their proxies.

It is also important to interpret survival data and mortality figures in the context of patients’ pre-existing frailty and limited life expectancy. It is estimated that 40% of patients 90 years or older who are admitted to a nursing home die within one year of admission. That number becomes 30% in patients of any age [3,25]. For those with advanced dementia, six-month mortality rates range between 24% and 37% [3,25,26]. These findings pose reasons to suggest that the higher mortality rates in those who choose nonoperative management for hip fractures do not necessarily indicate treatment failure. Rather, patients who decide against surgery may simply have poorer baseline health and prognoses [27,28]. 

Patients who are in the final stages of life may value alternative treatments to surgery for various reasons. As such, this requires a change of perspective within the healthcare community regarding what constitutes successful and unsuccessful management of hip fractures.

Quality of life

Surgical intervention is often required for several types of lower extremity fractures, particularly those of the hip [4,29]. Operative management of hip fractures is important for improving mobilization and, in certain cases, to prevent or resolve severe complications, such as avascular necrosis of the femoral head. These interventions also seek to improve quality of life (QOL) through restoring mobility. Helping patients regain their mobility and return to their pre-fracture functional status is of particular importance in the older adult population [30,31]. While surgery generally improves mobility, there are rare cases in which it may cause periods of prolonged immobilization. In a study by Siu et al, longer periods of immobility following surgical treatment for hip fracture were associated with poorer function at two months and a higher six-month mortality [31].

Despite the evidence that postoperative mobilization improves morbidity in older hip fracture patients, the percentage of patients actually regaining baseline mobility is considerably low. The FRAIL-HIP study found that among patients who were mobile before their injury, only 29% returned to some level of mobility following surgical intervention during the approximately two-year study period [3]. Additionally, mortality rates in older hip fracture patients are largely dependent on pre-injury mobility status. Those who are mobile before surgery tend to have better outcomes; whereas, higher mortality rates are more strongly associated with those who have less mobility independence and require assistance or supervision [31]. Thus, any increase in QOL that can be gained by returning to mobility appears to be experienced by only a small proportion of this population. As such, it calls into question the potential for positive impact that hip fracture surgery has on older patients who have limited or no mobility before their injury.

As of July 2022, approximately 1.2 million people reside in nursing homes in the United States [32]. The majority of this population consists of individuals who are quite frail and receiving end-of-life care services [33]. When caring for those nearing the end of life, a major focus is on ensuring comfort and optimal QOL [34]. For these patients, surgical intervention for certain hip fractures may not always be the best option. It is important to remember that QOL is a subjective matter and does not necessarily equate with prolongation of life. There is evidence to suggest that nonoperative treatment can indeed provide comparable or perhaps even better QOL outcomes for select older adults [35]. The FRAIL-HIP study used the EuroQol 5-Dimension 5-Level (EQ-5D-5L) utility score, assessed by proxies and caregivers, to determine that nonoperative treatment provided a comparable QOL to operative treatment for proximal femoral fractures in institutionalized frail older patients with limited life expectancy [3]. Researchers in this study found that mortality was understandably higher in the nonoperative group; however, there was no decrease in QOL, and the ratings for treatment satisfaction and humane quality of dying were high [3]. This evidence suggests that healthcare providers should not necessarily jump to surgery as the primary treatment in older adults receiving palliative care, hospice, or other end-of-life services. These patients often have extensive and severe comorbidities. This requires healthcare providers to facilitate a shared decision-making process with patients and their families to properly treat hip fractures and manage expectations regarding good QOL, bearing in mind that surgery may not always be the best option [3]. A comprehensive assessment followed by a proper shared decision-making process may even result in more patients choosing nonoperative management [12].

Older adults who choose to forgo operative management for hip fractures may elect to pursue earlier palliative care treatment. At this point, the main priority would be to control pain and prevent suffering, which aligns with the goals of palliative medicine [3,36]. Additionally, palliative care physicians are specifically trained to coordinate care and remain in constant communication with patients and their families [36]. Early integration of palliative care can also improve QOL by not only addressing physical symptoms, but also attending to psychosocial and spiritual concerns [4,6]. Altogether, these actions are meant to improve a patient’s QOL by shifting the focus from prolonging life to maximizing comfort and quality of their remaining life.

Harm versus burden

The task of taking care of patients at the end of life can be very difficult, especially for patients who have multiple comorbidities [37]. When considering the goals of palliative care for patients with hip fractures, it is crucial to understand the difference between harm and burden [38]. Harm is a measure of the direct negative outcomes resulting from an intervention, whereas burden is a measure of the overall impact of a treatment plan on a patient's quality of life [38]. For example, subjecting a patient to highly toxic chemotherapy is considered harmful because it causes direct physical damage, whereas requiring a patient to undergo numerous inconvenient doctor visits for essential care is a burden because it causes a disruption of routine and often discomfort.

When considering older frail patients, and especially patients with neurocognitive disabilities, operative procedures can cause significant harm by increasing the likelihood of complications, deteriorating cognition, and longer hospital stays [9,10]. While some patients may prefer the burden of interventions in an effort to prolong life, the potential harm of surgery and subsequent rehabilitation may outweigh the benefits, particularly for patients with limited life expectancy [11,39].

In contrast, when considering nonoperative management, although short-term mortality rates are often higher, this option may better align with a patient’s goals of care, especially if the patient has a poor prognosis and desires to prioritize QOL and comfort in their final days [3,40]. Conservative treatment can help control pain and make patients more comfortable, while also reducing the likelihood of complications due to surgical procedures, thus improving their QOL [41]. By performing a harm versus burden assessment for each individual case, patients and their families can decide on a treatment plan that fits the patient’s individual needs and desires.

Limitations

This study has several limitations that should be noted. There are various frailty assessment tools that have been used in studies cited in this paper; thus, it is difficult to compare and contrast the outcomes of some of the studies, as their metrics had differences [42,43]. As such, there is a need for a standardized assessment tool that can be used to assess frailty so that the various data sets can be accurately compared. In addition, the quality of life metric is subjective in nature, which makes it more difficult to properly evaluate treatment outcomes [44].

Furthermore, there is likely a degree of selection bias in the data comparing patient outcomes in operative versus nonoperative interventions since patients were not randomly assigned to the two groups. It is possible that patients with worse prognoses chose nonoperative management plans more often than patients with better prognoses, thus creating bias within the data on mortality rates [3]. Additionally, a randomized controlled study on this issue would not be feasible due to ethical concerns [3]. It is also important to acknowledge that the data regarding quality of life is often gathered from patient proxies, which can introduce bias since these reports may reflect proxies' perceptions and hopes rather than patients' direct experiences. Lastly, considering the nature and condition of patients in various studies, follow-up time was comparably short in many cases. This limits the ability to extrapolate findings to long-term outcomes [3].

Summary of findings

This review compared operative and nonoperative treatment for hip fractures in the frail older adult population. Both treatment routes were evaluated with a focus on quality of life outcomes, survival, and harm. Herein, we propose that nonoperative management can be effective, and sometimes preferred for certain frail patients on a palliative care or hospice service with less than six months of life expectancy. Although surgery remains the standard of care for most hip fractures, emerging evidence suggests that nonoperative approaches may provide some patients with comparable or even improved quality of life [3,4,40,45].

Surgical intervention for certain hip fractures has been shown to decrease mortality and improve mobility in some older patients [3]. However, it is well known that surgery and the administration of anesthesia carry risks of side effects and complications [9,10]. As stated by the National Hospice and Palliative Care Organization, palliative care optimizes quality of life by anticipating, preventing, and treating suffering [46]. Proxies of those living in nursing homes agree that only treatments that promote comfort should be pursued, thus implying that operative management may not always align with the goals of care for palliative patients [3,21]. The nonoperative management of hip fractures, in conjunction with palliative care, can ensure respect for patients’ end-of-life preferences while prioritizing comfort both physically and emotionally [4,41]. Table 1 summarizes the most relevant studies on operative and nonoperative management of hip fractures in older adults.

**Table 1:** Comparison of key findings regarding operative versus nonoperative management of hip fractures in frail older adults.

Studies	Study/article focus	Key finding or contribution
Loggers et al. (2022)	Comparison of operative versus nonoperative management in frail institutionalized patients with proximal femoral fractures (FRAIL-HIP study).	Nonoperative treatment was linked to higher mortality but provided comparable quality of life (QOL) and higher satisfaction with the quality of dying compared to operative management in this patient group.
Sullivan et al. (2019)	Palliative care in the hip fracture patient.	Emphasizes the importance of palliative care integration for hip fracture patients, especially in complex cases, noting that nonoperative management may align with patient goals.
Tremblay et al. (2024)	Scoping review of strategies to improve end-of-life decision-making and palliative care following hip fracture.	Highlights nonsurgical management involving pain control and early mobilization and the need for improved end-of-life decision-making and palliative care integration.
Joosse et al. (2019)	Protocol for the FRAIL-HIP multicenter observational cohort study.	Outlines the rationale for studying nonoperative versus operative treatment in frail institutionalized patients, focusing on the value of conservative management in the "shade of life."
Berry et al. (2018)	Association of clinical outcomes with surgical repair versus nonsurgical management in nursing home residents with advanced dementia.	Suggested that nonsurgical management may be a more appropriate course for nursing home residents with advanced dementia compared to surgical repair.
Siu et al. (2006)	Effects of early ambulation after hip fracture on function and mortality.	Found that early ambulation after hip fracture surgery is associated with improved function and lower six-month mortality; pre-injury mobility predicts better outcomes.
Cowan et al. (2017)	Challenges of anesthesia and pain relief in hip fracture care.	Discusses the significant risks of anesthesia and pain relief in hip fracture surgery, especially for older patients with comorbidities.
Funder et al. (2009)	Anesthesia for the patient with dementia undergoing outpatient surgery.	Addresses the potential for additional impairment in cognitive function in patients with neurocognitive disorders who undergo surgery.
Neuman et al. (2014)	Survival and functional outcomes after hip fracture among nursing home residents.	Provides data on survival and functional outcomes for nursing home residents with hip fractures, highlighting the generally poor prognosis in this setting.
Moulton et al. (2015)	Outcome after conservatively managed intracapsular fractures of the femoral neck.	One of the studies supporting the viability of nonoperative management for specific hip fractures.
Loggers et al. (2020)	Systematic review and meta-analysis on the prognosis of nonoperative treatment in elderly patients with a hip fracture.	Concludes that higher mortality in nonoperative groups may be due to poorer baseline health and prognoses rather than treatment failure.
Eleff (2022)	Discusses the concept of harm versus burden in clinical decision-making.	Differentiates harm (direct negative consequences of intervention) from burden (overall impact on QOL) in care planning.
van der Zwaard et al. (2020)	Impact of adding a comprehensive geriatric assessment in older patients with a hip fracture.	Found that incorporating a comprehensive geriatric assessment led to fewer patients undergoing surgery, supporting a more individualized approach.

## Conclusions

This review highlights the complexity of caring for frail older adults with hip fractures. It underscores the need for further research to better identify the characteristics and variables that indicate when nonoperative management may be superior to surgical intervention. While surgical treatment remains an important option, nonoperative management should be recognized as a viable alternative for select frail older adults, particularly when aligned with palliative care goals that extend beyond physical recovery and include emotional and psychosocial well-being. The ideal treatment plan requires individualization, which occurs when healthcare providers facilitate shared decision-making and take into account factors such as frailty, cognitive status, pre-fracture functional level, and patient preferences. Patients should also be encouraged to discuss their values and goals of care with family members before an injury occurs, to make shared decision-making more effective should the situation arise. The considerations outlined in this review may serve to guide patients, families, and clinicians in these complex decision-making processes while also serving to inform future areas of research.
